# The Modulation of Compositional Heterogeneity for Controlling Shear Banding in Co-P Metallic Nanoglasses

**DOI:** 10.3390/nano14120993

**Published:** 2024-06-07

**Authors:** Tian Li, Nana Li, Tianlai Yu, Guangping Zheng

**Affiliations:** 1 CDGM Glass Co., Ltd., Chengdu 610199, China; 2 Department of Mechanical Engineering, The Hong Kong Polytechnic University, Hong Kong SAR 999077, China; 3 Chengdu Guangming Paite Precious Metal Co., Ltd., Chengdu 610199, China

**Keywords:** mechanical properties, glass-glass interfaces, metallic nanoglasses, elemental segregation, molecular dynamics

## Abstract

Shear banding is much dependent on the glass–glass interfaces (GGIs) in metallic nanoglasses (NGs). Nevertheless, the current understanding of the glass phase of GGIs is not well established for controlling the shear banding in NGs. In this study, Co-P NGs are investigated by molecular dynamics simulations to reveal the phenomenon of elemental segregation in the GGI regions where the content of Co is dominant. Specifically, Co segregation results in the formation of GGIs, whose atomic structures are comparatively less dense than those present in the interiors of glassy grains. It is suggested that the Co segregation significantly reduces the shear resistance of GGIs. Thus, such compositional heterogeneity influences the mechanical properties of Co-P NGs. Particularly, shear banding is much altered through enhancing the Co segregation in the GGI regions, which leads to improvements in the ductility of Co-P NGs. This study advances knowledge of the formation of the GGI phase in NGs, which could enable GGI engineering in enhancing the mechanical properties of NGs.

## 1. Introduction

Metallic glasses (MGs) are fully disordered in their crystal structures [1], and the absence of dislocation defects leads to the remarkable mechanical strength of MGs [2,3,4], making them ideal candidates for structural applications in engineering [5,6]. However, the applications of MGs are still limited up until now, mainly due to the intrinsic brittleness issues arising from the formation of predominant shear bands when they are under plastic deformation [7,8,9]. Over the last several decades, lots of efforts [10,11,12,13] have been made to control shear banding and to achieve decent ductility in MGs [14,15,16,17,18]. Previous research work [19,20,21] suggests that the MG-based composites are feasible for preventing the localization of plastic deformation and shows that shear banding is reliant on the physical properties of the second phases in MG-based composites.

Recently, a new type of MG-based composites, namely, metallic nanoglasses (NGs), have been successfully prepared [22,23,24], which consist of glassy grains separated by the glass–glass interfaces (GGIs). Based on the dual-phase model suggested for those NGs [25,26,27,28,29], the glassy grains are analogous to those rapidly-quenched MGs with nano-sized dimensions [30,31]. In contrast, the GGIs, which have been determined to possess unique atomic structures, could be with a new glass phase [32,33,34,35,36,37,38], i.e., the second phase in NGs. More importantly, it has been found that the growth of major shear bands can be suppressed to overcome the brittle fractures in NGs [39]. For instance, Adibi et al. [40,41] demonstrated the capability of shear bands in NGs to be uniformly distributed within the GGI regions, which was further explained by Wang et al. [42], who demonstrated that the GGIs would promote the plastic deformations in NGs. In other words, the GGIs could significantly influence the shear banding in NGs [43,44,45,46,47,48], although a viable strategy for controlling shear banding through alternating the glass phase of GGIs is yet not available, which is critical to further improving the ductility of NGs.

Recent studies reported the elemental segregation of GGIs that leads to the compositional heterogeneity observed in NGs. In the GGIs in Cu-Zr NGs, for instance, there are increasing and decreasing numbers of Cu and Zr atoms [49,50]. Similarly, an Fe segregation was characterized for the GGI regions in Fe-Sc NGs [51]. However, research on the relationship between shear banding in NGs and compositional heterogeneity in GGIs is scarce. In our previous work [36,37,38,47,52], we demonstrated that the (Co, P)-based NGs could be synthesized through pulse electrodeposition, although the fabrication of bulk NGs through pulse electrodeposition is still challenging, which prevents us from further conducting an experimental study on investigating their mechanical properties. In this paper, Co-P NGs for MD simulations are developed to resolve the aforementioned concerns. The Co segregation is observed in the GGI regions, where the excess free volumes resulting from Co segregation are evident. Consequently, upon comparison with the interiors of glassy grains, the atomic structures of GGIs are suggested to be less dense. Moreover, the presence of compositional heterogeneity significantly influences the shear banding in Co-P NGs, indicating that it is a viable approach for enhancing the ductility of NGs.

## 2. Simulation Methodologies

The large-scale atomic/molecular massively parallel simulator was employed to create MD simulations [53]. The Pak–Doyama-type pair potential was used to characterize the interatomic potentials between Co-P, Co-Co, and P-P [54]. The following are the possible representations of potential energy (E_ij_) measured in eV between atoms i and j:

E_Co-Co_(r) = −0.12812(r − 1.82709)^4^ + 1.15421(r − 2.50849)^2^ − 0.13448, r < 3.34 Å (1)

E_Co-P_(r) = −0.15374(r − 1.58709)^4^ + 1.38505(r − 2.26849)^2^ − 0.13167, r < 3.20 Å (2)

E_P-P_(r) = −0.07435(r − 2.60709)^4^ + 0.64791(r − 3.27885)^2^ − 0.07531, r < 4.21 Å (3)

in which r represents the separation of atoms i and j. When r was larger than the cutoff distance, E_ij_ was set to 0 eV. The reliability of the interatomic potentials used for simulating the mechanical properties of Co-P NGs was demonstrated by the fact that the amorphous atomic structures of the Co-P glasses obtained from the MD simulations are well corresponded with those observed in the experiments [55]. Implementing the conditions of periodic boundary along the x-, y-, and z-axes, the equations of motion were integrated numerically with 1 fs timestep. The Barostat and Nose–Hoover thermostat were respectively employed to regulate the pressure and temperature of the simulated supercells.

The Co_x_P_100−x_ (70 ≤ x ≤ 90) alloy with a crystalline structure was developed [56] and then allowed to relax at a temperature of 300 K using an isobaric–isothermal ensemble. Initially, the temperature of the relaxed crystalline Co-P alloy was maintained at a temperature of 2000 K until its complete phase transition to liquid. Following this, the melts were quickly cooled to 300 K at a 10^10^ K/s rate, yielding Co_x_P_100−x_ (70 ≤ x ≤ 90) MG, which demonstrated a well-defined glassy phase. Following this, the Co_x_P_100−x_ (70 ≤ x ≤ 90) MG was cut into a bar with a 25 nm diameter and a height-to-diameter aspect ratio of 2:1, which was orientated in the z-direction. It was further cut in half at an angle of 30° with regard to the x-direction, resulting in two MG blocks that were separated by 10 nm, and formed two free surfaces. After heating the MG blocks to 400–550 K for 20 ns to bring their free surfaces to an equilibrium state, they were bonded together using the interdiffusion of atoms across their relaxed surfaces. This process was carried out under a constant pressure of 1 bar applied along the z-axis, and then the blocks were cooled to 300 K. Consequently, a nano-bar containing two MG blocks separated by one GGI was obtained.

The MGs were filled into a nanocrystalline model, whose nanostructures, including grain shapes, sizes, and interfaces, as well as triple junctions among the grains, could be fine-tuned to mimic the experimental results, allowing for the construction of nano-pillars containing glassy grains of varying sizes [57]. The construction process consisted of the following procedures: First, as described previously, atoms in the Co_80_P_20_ MG model, which was generated via MD simulation, were initially filled into the regions defining the grains’ interiors in the nanocrystalline model. This resulted in the formation of NG models comprising glassy grains with sizes that varied over a range of 32 nm. Second, in order to form NG models accompanied by well-separated glassy grains, the distances between surface atoms traversing the glassy grains were limited to a minimum of 2 nm, preventing the adjacent glassy grains from being very close to one another. Subsequently, the NG models were cut into nano-pillars with 25 nm diameters and a height-to-diameter aspect ratio of 2.4:1, which stood in the z-direction. Furthermore, in order to achieve free surface equilibration across the glassy grains in the systems, the nano-pillars were maintained at 500 K for a duration of 20 ns in the MD simulation. Subsequently, the nano-pillars were compacted in the z-direction under a pressure of 1 bar and were cooled to 300 K. Ultimately, nano-pillars incorporating diverse GGIs were obtained. This work reports the construction of nano-pillars with different average sizes of glassy grains, i.e., D_avg_ = 5, 7.5, 10, and 20 nm, for the process of MD simulations and visualized by the OVITO software package (Version 3.0.0) [58].

## 3. Results and Discussion

**Figure 1:**
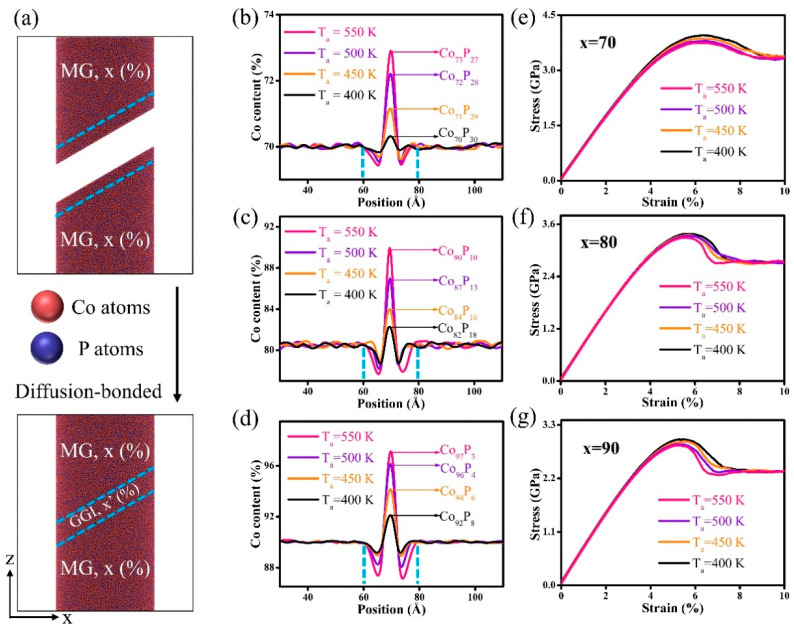
(**a**) Schematics of the formation of a nano-bar system containing one GGI in the MD simulations; the atoms in the GGI region are represented by dash lines. The concentrations of the elements in the systems with compositions of (**b**) Co_70_P_30_, (**c**) Co_80_P_20_, and (**d**) Co_90_P_10_; the width of GGIs is indicated by the dash lines. The obtained curves of stress vs. strain for the systems with compositions of (**e**) Co_70_P_30_, (**f**) Co_80_P_20_, and (**g**) Co_90_P_10_.

The system started with two Co_x_P_100−x_ MG blocks (70 ≤ x ≤ 90) with identical compositions, indicated in Figure 1a, where x represents the quantity of Co present within the MG blocks. These two MG blocks were initially maintained at an annealing temperature, i.e., T_a_ = 400–550 K (glass transition temperature, T_g_~550 K), to relax the free surfaces (as marked by dash lines) and then were diffusion-bonded across the relaxed free surfaces, resulting in a nano-bar containing one GGI locating between two MG blocks. In this study, T_g_ is identified at the onset temperature of the abrupt decrease in density of the system during the quenching process. The T_a_ was chosen in the range of 0.7 T_g_ < T_a_ < T_g_ to facilitate the formation of GGIs in systems without crystallization transformation. The compositions of the GGIs were analyzed by probing the atomic concentration profiles, and the data presented in Figure 1b–d manifest the fact that the compositional heterogeneity resulting from Co segregation at the interfaces was strongly influenced by T_a_. For example, after T_a_ was increased from T_a_ = 400 to 550 K, the content of Co (x’ at the center of GGI region) increased from x’ = 70 to 73 when x = 70. Such Co segregation in the GGIs was more pronounced in the systems with compositions of Co_80_P_20_ and Co_90_P_10_, and a variation in the content of Co x’ from x’ = 82 to 90 and from x’ = 92 to 97, respectively, though annealing could be dramatic when x = 80 and 90, respectively. The dependence of the Co content x’ on the chemical composition (x) demonstrates that the compositional heterogeneity at GGIs were much affected by the interiors of the MG blocks. The variations in the compositional heterogeneity could potentially be accounted for by the differences in the mobility of the elements Co and P that were in close proximity to the unbound surfaces of the MG blocks during annealing.

**Figure 2:**
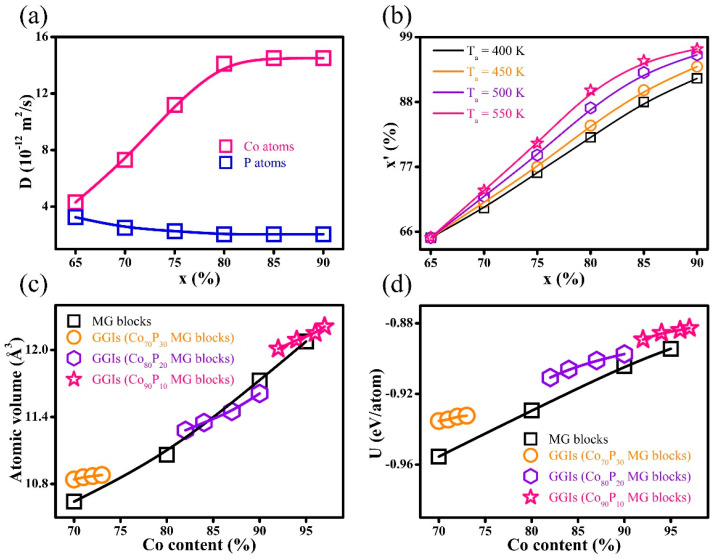
(**a**) Co and P atom diffusion coefficients D at the free surfaces of the MG blocks (T_a_ = 500 K). (**b**) The GGI compositions x’ determined in the systems annealed at T_a_ = 400, 450, 500, and 550 K. (**c**) The atomic volume and (**d**) atomic internal energy U for GGIs that had a content of Co of x’ and a Co content of x bearing MG blocks.

In general, the diffusivity of the element Co was greater than that of the P atoms. Moreover, as shown in Figure 2a, there were increases and decreases in the diffusion coefficients (D) for Co and P, respectively, with increasing Co content x in the interiors of MG blocks. Therefore, an increase in the Co content x of MG blocks significantly caused a greater diffusion of the Co atoms in the GGI regions as compared to the P atoms. Consequently, the Co segregation in GGIs was prominent in the systems with compositions of Co_80_P_20_ and Co_90_P_10_. Similarly, the D of element Co increased with increasing T_a_, resulting in a higher number of Co atoms occupying the free-volume sites in the GGI regions (Figure 2b). The fast diffusivity determined for Co atoms may be explained in view of atomic bond dissociation energy. Specifically, it is speculated that the dissociation energy of the Co-P bond is much improved as compared to Co-Co and P-P bonds, meaning that it requires more energy to break the Co-P bond for the atoms to diffuse. Considering the dominance of the element Co in the systems, most P atoms would prefer to bond with adjacent Co atoms, i.e., a Co-P bond, and the rest of the Co atoms would bond with other Co atoms, leading to the formation of a Co-Co bond. During annealing, there was a lack of a P-P bond, while the Co-Co bond was easier to break than the Co-P bond, resulting in Co atoms with fast diffusivity that were segregated in the GGI regions. Figure 1e–g illustrates the stress vs. strain curves of nano-bars under uniaxial compression, and the conditions of periodic boundary are applied in the z-axis. At a strain rate of 3 × 10^7^ s^−1^, the mechanical strength of systems with a composition of Co_70_P_30_ decreased with an increase in the compositional heterogeneity, as demonstrated by a higher Co content x’ in the GGIs. Similarly, the mechanical strength decreased with increasing Co content x’ in the GGIs in the systems with compositions of Co_80_P_20_ and Co_90_P_10_, demonstrating that the mechanical strength was reduced in Co-P NGs as compositional heterogeneity increased. As illustrated in Figure 2c, an increase in the Co content x’ at the center of the GGIs or x within the interiors of the MG blocks resulted in a corresponding increase in the free volume. Therefore, Co segregation produced the excess free volumes in the GGI regions, resulting in less dense atomic structures in the GGIs as compared to the block interiors. According to composition-dependent atomic internal energy (U) demonstrated in Figure 2d, the Co-P MGs were at a higher level with an increase in the Co content. Thus, the GGIs could be thermodynamically unstable in the system, as compared to the block interiors, simply because the Co segregation in the GGIs resulted in a Co content x’ higher than x.

**Figure 3:**
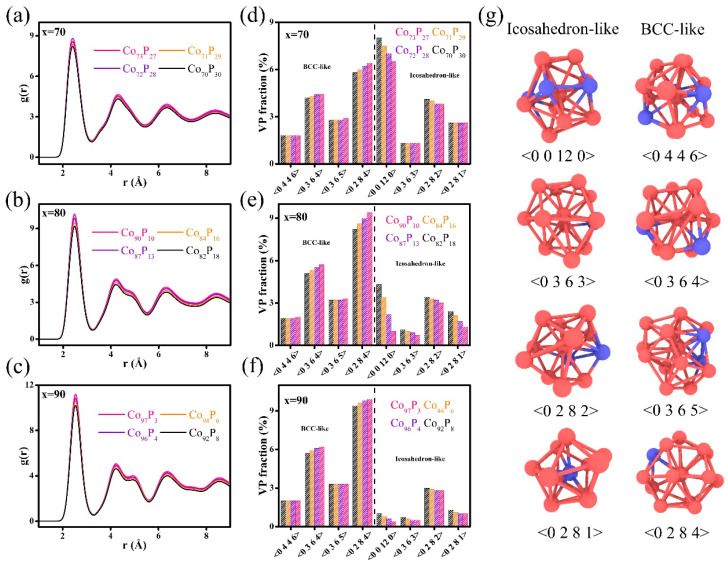
The RDFs for GGIs with various Co contents x’ in the systems with compositions of (**a**) Co_70_P_30_, (**b**) Co_80_P_20_, and (**c**) Co_90_P_10_. The VP fractions of GGIs with various Co contents x’ in the systems with compositions of (**d**) Co_70_P_30_, (**e**) Co_80_P_20_, and (**f**) Co_90_P_10_. (**g**) The schematic illustration of bcc- and icosahedron-like VPs.

The determined radial distribution functions (RDFs) for the GGIs are illustrated in Figure 3a–c. For the GGIs, an increase in Co content x’ typically resulted in higher peak intensities of RDFs. This characteristic was further confirmed by analyzing the topological atomic structures of GGIs, as described by Voronoi polyhedrons (VPs) shown in Figure 3d–f. Employing the Voronoi index, <n_3_ n_4_ n_5_ n_6_>, a VP comprised of n_3_, n_4_, n_5_, and n_6_ faces with respective edges of 3, 4, 5, and 6, was identified. It is important to note that the icosahedron-like VPs and the body-centered cubic (bcc)-like VPs have respective coordination numbers (n_3_ + n_4_ + n_5_ + n_6_) of 11–12 and 13–14. These coordinate numbers predominated in the entire VP population, as presented in Figure 3g. It can clearly be seen that the bcc-like VPs proportions in the GGI regions increased as the content of Co x’ increased. The increased peak intensities of RDFs may be attributable to the increased proportion of bcc-like VPs, and the GGIs with a greater abundance of bcc-like VPs are thought to be less disordered. However, the icosahedron-like VPs fractions at the GGIs were reduced after the Co content x’ was increased. It has been established that amorphous alloys with a smaller amount of icosahedron-like VPs demonstrate a decreased shear resistance as well as mechanical strength, rendering them more prone to plastic deformation under externally applied stresses [59,60,61,62]. Therefore, the mechanical strength of nano-bars decreased with increasing compositional heterogeneity, i.e., by increasing the Co content x’ in the GGIs, which was largely attributed to the decrease in the proportions of icosahedron-like VPs in the GGIs. Therefore, compositional heterogeneity could significantly influence the mechanical properties of Co-P NGs.

**Figure 4:**
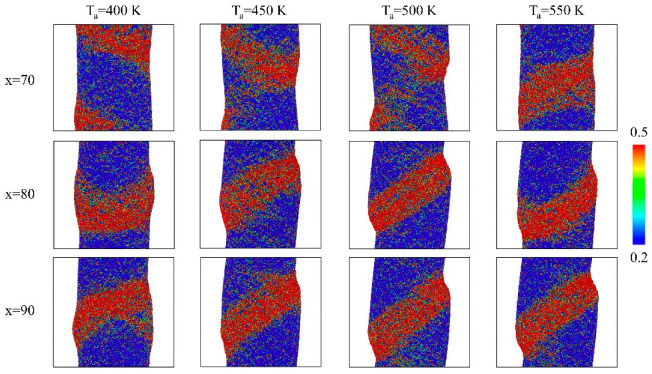
The atomic shear strain mappings in systems with compositions of Co_70_P_30_, Co_80_P_20_, and Co_90_P_10_, under an applied strain of 10%; the atoms with a local shear strain equal to 0.2 or greater have been highlighted.

The shear bands within the nano-bars containing one GGI are illustrated in Figure 4. The shear transformation zone (STZ), i.e., the embryonic shear band, is a region characterized by an atomic shear strain of 0.2 or greater under plastic deformation. Its precise definition can be written as follows [59,63,64]:

(4) $$\eta_{i}=(J_{i}J_{i}^{T}-I)/2$$

(5) $$\eta_{i}^{Mises}=\sqrt{\eta_{xy}^{x}+\eta_{xz}^{2}+\eta_{yz}^{2}+{\left[(\eta_{xx}-\eta_{yy}\right)}^{2}+{\left(\eta_{xx}-\eta_{zz}\right)}^{2}+{\left(\eta_{yy}-\eta_{zz}\right)}^{2}]/6}$$

where J_i_ denotes the local transformation matrix for atom i and $\eta_{i}$ represents the local Lagrange strain matrix for atom i, while $\eta_{i}^{Mises}$ refers to the local shear strain for atom i. Taking the systems with a composition of Co_70_P_30_ as an example, the embryonic shear bands mainly initiated and propagated inside the MG blocks processed by annealing at T_a_ = 400 K. Nonetheless, there was a transition for the localization of STZs in the interiors of the MG blocks to the GGI regions when T_a_ increased from T_a_ = 400 to 550 K, indicating that the GGIs with increased Co segregation were more favorable for the shear banding, which can be attributed to the smaller shear resistance of the GGIs caused by a lower fraction of icosahedron-like VPs. Consequently, the embryonic shear bands were prominent across the GGIs in the system processed by annealing at T_a_ = 550 K. For systems with compositions of Co_80_P_20_ and Co_90_P_10_, the STZs primarily localized within the GGI regions (processed by annealing at T_a_ = 400 K), simply because the Co segregation in the GGIs was more significant in the systems with Co contents of x = 80 and 90. Furthermore, an increase in the number of embryonic shear bands at GGIs resulting from an increase in the Co content x’ was observed and, as a result, the shear banding could be better restricted within the GGI regions in the systems with enhanced compositional heterogeneity. Therefore, it is evident that the modulation of compositional heterogeneity is a feasible approach for controlling the shear banding in Co-P NGs undergoing plastic deformation.

**Figure 5:**
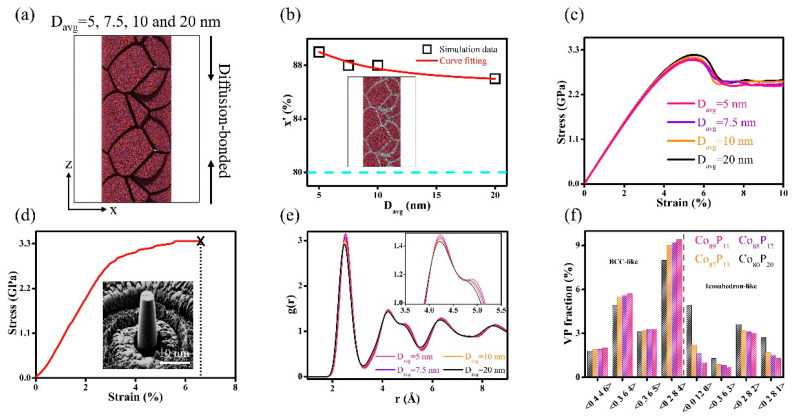
(**a**) Schematics of the MD simulations illustrating the formation of nano-pillars comprised of glassy grains of varying sizes. (**b**) Size effects on the chemical composition of the GGIs (black symbols represent the x’); the dashed line denotes (x-80) in the glassy grain interiors. (**c**) Stress vs. strain curves of nano-pillars with different D_avg_. (**d**) Pillar compression tests on Co-P NGs with D_avg_~67 nm (experiments); the inset shows the image on the pillars. (**e**) The effects of D_avg_ on the RDFs of the nano-pillars. The magnified image of the second RDF peak is shown in the inset. (**f**) The fractions of the VPs within the GGI regions with different chemical compositions (x’ = 89, 88, and 87) and in the Co_80_P_20_ glassy grain interiors.

The nano-pillars containing glassy grains with different D_avg_ were further constructed to investigate the impact of compositional heterogeneity on shear banding in Co_x_P_100−x_ NGs, as visualized in Figure 5a. These systems are comprised of glassy grains with D_avg_ = 5, 7.5, 10, and 20 nm, with a fixed chemical composition of x = 80. After maintaining nano-pillars at a constant temperature of 500 K, they were diffusion-bonded across the free surfaces of adjacent glassy grains, resulting in the emergence of regions having two different glassy phases in the nano-pillars, i.e., the regions of GGIs containing x’ and the interiors of glassy grains containing x = 80 (the inset in Figure 5b). Herein, the mean Co content in the GGI regions is referred to as x’. An increase in Co content x’ through reducing D_avg_ is illustrated in Figure 5b, suggesting that the compositional heterogeneity depends on the size of glassy grain, which implies that reducing the D_avg_ can increase the amount of Co segregated in the GGI regions. The stress vs. strain curves of the nano-pillars under uniaxial compression are shown in Figure 5c. The decrease in mechanical strength with increasing Co content x,’ as caused by a lower D_avg_, was consistent with the aforementioned findings, indicating that the Co-P NGs with more compositional heterogeneity tended to have lower mechanical strength, which can be directly compared with the experimental results. We have previously shown that the Co-P NGs containing Co_80_P_20_ glassy grains with D_avg_~67 nm can be prepared from pulse electrodeposition [52]. In the pulse electrodeposited Co-P NGs, the x’ of the GGIs is characterized as x’ = 85. Figure 5d illustrates the pillar compression tests on pulse electrodeposited Co-P NGs, whose mechanical strength was determined to be 3.36 GPa when x’ of the GGIs was x’ = 85. It is worth noting that the mechanical strength of the nano-pillars was determined to be 3.05, 3.11, and 3.18 GPa by MD simulations when x’ of GGIs was x’ = 89, 88 and 87, which were in good agreements with the experimental results, further demonstrating that the mechanical strength of Co-P NGs decreased with increasing compositional heterogeneity. The changes in compositional heterogeneity by D_avg_ may be well reflected by the RDF analyses shown in Figure 5e. In general, a decreased D_avg_ resulted in greater peak intensities for RDFs. The increased intensity, specifically observed at the shoulder of the second RDF peak, absolutely signified the new glassy phase formation. Due to the fact that compositional heterogeneity increased as D_avg_ decreased, it is hypothesized that the atomic structures of the GGIs, which having greater x’, are significantly distinct from those found in the glassy grain interiors. Figure 5f presents the analyses of the topological atomic structures at the GGI regions as well as in the interiors of glassy grains. There was a respective increase and decrease in the fractions of the bcc- and icosahedron-like VPs within the GGI regions, which had comparatively increased contents of Co x’ as compared to the interiors of glassy grains with a Co content of x = 80.

**Figure 6:**
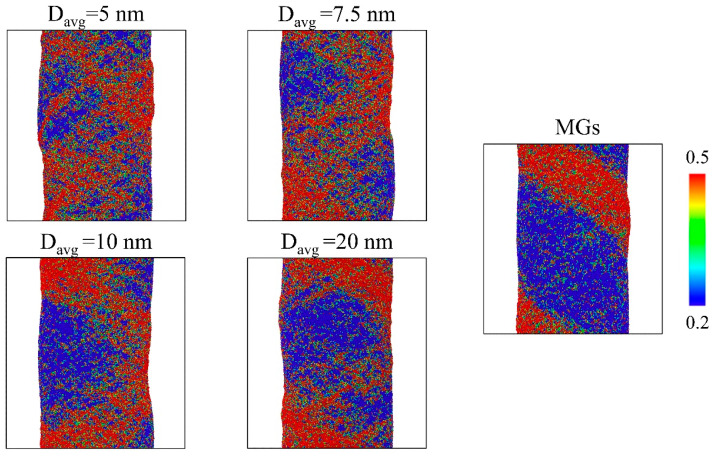
The atomic shear strain mappings on nano-pillars containing glassy grains with D_avg_ = 5, 7.5, 10, and 20 nm and on nano-pillars without GGIs under an applied strain of 10%; the atoms with local shear strains either greater than or equal to 0.2 have been highlighted.

The shear banding in the nano-pillars with D_avg_ = 5, 7.5, 10, and 20 nm, and in the nano-pillars without GGIs (MGs) under an applied strain of 10% are illustrated in Figure 6. Obviously, the nucleation of STZs at the ends of the nano-pillars without GGIs could be significant, resulting in predominant shear bands that could lead to catastrophic brittle fractures. In contrast, shear bands were less localized at the ends of nanostructured nano-pillars with D_avg_ = 20 nm, resulting from the formation of GGIs. When D_avg_ was further reduced to D_avg_ = 10 and 7.5 nm, respectively, the shear bands were more uniformly distributed in the systems because they were enabled to propagate into the body of nano-pillars. Moreover, a relatively homogenous plastic deformation was observed in the systems containing glassy grains with an extremely small D_avg_ = 5 nm, providing evidence of reasonable enhancements in the ductility. Generally, the GGIs with enhanced Co segregation had lower shear resistance and thus promoted shear banding, i.e., plastic deformation, in the GGI regions. For Co-P nano-pillars with an increased D_avg_, the GGIs had a reduced Co segregation. Therefore, such GGIs possessed atomic structures similar to those found in the interiors of glassy grains. The characteristics of shear banding in these Co-P nano-pillars resembled those observed in the MGs lacking GGIs, which displayed brittle fractures and inhomogeneous plastic deformation. For Co-P nano-pillars with a smaller D_avg_, the GGIs with much higher Co content x’ compared with (x) in the interiors of glassy grains were activated for shear–band propagation and the global existence of GGIs with a significantly enhanced Co segregation would eventually cause the plastic deformation to be homogenous, which is much different from the localized plastic deformation, as observed in the brittle nano-pillars without GGIs. Therefore, it is the compositional heterogeneity that alternates the shear banding, as observed in Figure 6, which could be beneficial to improving the ductility of nano-pillars.

## 4. Conclusions

In summary, the effects of compositional heterogeneity were investigated on the shear banding in Co-P NGs. The segregation-based compositional heterogeneity was determined within the GGI regions, whose atomic structures as altered by the segregation of the element Co could be less dense as compared with the glassy grains’ interiors. Furthermore, the Co segregation in GGIs was strongly associated with the shear banding in Co-P NGs under plastic deformation. It was determined that the GGIs showing enhanced Co segregation would facilitate shear banding. Therefore, Co-P NGs with increasing compositional heterogeneity are suggested to have better capability of preventing the localization of shear bands and, thus, achieving a homogenous plastic deformation, which would lead to improvements in the ductility of NGs.

## Data Availability

The datasets that were generated and/or analyzed during this study are available from the corresponding author upon reasonable request.
